# Challenges and dynamics in reporting medical device incidents: a qualitative study

**DOI:** 10.3389/frhs.2025.1720494

**Published:** 2025-12-10

**Authors:** Meital Mishali, Nadav Sheffer, Maya Negev

**Affiliations:** 1 The Health and Climate Resilience Lab, School of Public Health, Faculty of Social Welfare and Health Sciences, University of Haifa, Haifa, Israel; 2 School of Medical Engineering, Afeka—The Academic College of Engineering, Tel Aviv, Israel

**Keywords:** adverse event, medical device, post market surveillance, reporting, stakeholders

## Abstract

**Background:**

Adverse event reporting for medical devices is essential for post-market surveillance and public health, preventing harm like patient injury, misdiagnosis, or death and is shaped by regulation that defines policies, enforcement, and responsibilities. Various factors—poor maintenance, manufacturing defects, user error, or clinical issues—can cause adverse events, complicating root cause identification, sometimes leading to ambiguous accountability and hindering prevention.

**Objective:**

To examine perceptions, behaviors, and challenges in incident reporting among key stakeholders in the public and private sector in Israel, as a case study for a country where a Medical Devices Law is not yet effective.

**Methods:**

A qualitative thematic analysis was conducted based on 31 in-depth interviews with stakeholders from the Ministry of Health, healthcare institutions (e.g., physicians, nurses, management), and medical device companies (e.g., CEOs, regulatory affairs managers). Interviews were transcribed and coded using inductive thematic analysis.

**Results:**

Four key themes emerged. First, the complexity of causality in device-related events often shifted responsibility between device manufacturer and user, complicating root cause identification and accountability. Second, communication among stakeholders was often described as complex and unclear, sometimes influenced by conflicting interests. Third, reporting behavior was shaped by organizational culture, particularly management's attitude, which could foster or suppress engagement. Nurses were generally more active reporters than physicians. Fourth, a lack of feedback was a recurring concern, reducing motivation to report.

**Conclusion:**

Barriers to reporting include regulatory gaps, unclear procedures, communication challenges, and legal concerns. Still, many participants described positive inter-organizational collaboration. Stronger regulation and feedback mechanisms, clearer role definitions, particularly between physicians and nursing staff, and supportive managerial attitudes may foster a more responsive reporting culture. By incorporating diverse stakeholder perspectives, this study highlights lessons of broader relevance for improving medical device vigilance and patient safety worldwide.

## 1 Introduction

Medical devices play a critical role in modern healthcare, supporting diagnosis, treatment, monitoring, and patient safety across a wide range of clinical contexts (1). However, their use is not without risk. Adverse events involving medical devices—ranging from device malfunction to misuse or patient harm—pose significant threats to quality of care and public health. To mitigate these risks, post-market surveillance systems rely heavily on the timely and accurate reporting of such events by healthcare professionals, institutions, and medical device companies.

Despite global recognition of the importance of reporting for regulatory oversight and patient safety, underreporting remains a persistent challenge. Studies across diverse healthcare systems have shown that many adverse events go undocumented or unreported, limiting the ability to detect patterns, identify risks, and implement preventive measures (2, 3). Barriers to reporting may include uncertainty about what constitutes an adverse event, lack of awareness of reporting procedures, fear of blame, organizational culture, or perceived inefficacy of the reporting system (4–7). Moreover, reporting systems differ in structure and implementation across countries, influenced by local regulations, organizational practices, and stakeholder roles. In many settings, limited systemic data collection results in reliance on voluntary reporting by individual clinicians—reducing the consistency and completeness of available information (8).

In the European Union, the Medical Device Regulation 2017/745, which came into force in 2021 (9), introduced more stringent obligations for manufacturers, importers, and authorized representatives, including centralized reporting and proactive post-market surveillance (10). In the United States, the Food and Drug Administration (FDA) requires manufacturers and user facilities to report specific device-related incidents within defined timeframes (11). These reports are compiled in the publicly accessible Manufacturer and User Facility Device Experience (MAUDE) database (12), which serves as a key tool for identifying safety signals and monitoring trends (13, 14). Despite these efforts, underreporting remains a concern. Reports are often delayed, incomplete (15), or inconsistent, reflecting ongoing challenges in achieving effective post-market monitoring.

In Israel, regulatory oversight of post-market surveillance is structured differently from the systems established in the United States and European Union. Although a Medical Devices Law was enacted in 2012 (16), it has not yet been implemented and therefore does not impose binding vigilance obligations on manufacturers or hospitals. In practice, device vigilance operates de facto: registration holders and manufacturers are required—through licensing conditions and Ministry of Health guidance—to report adverse events and field safety actions to the Medical Device Division, which maintains the national database of adverse events and is responsible for their evaluation and follow-up. Following this evaluation, the Division assesses whether regulatory action is required, which may include revoking a device's marketing approval, coordinating a voluntary market recall with the manufacturer, or recommending enhanced user training for healthcare staff. Healthcare institutions, by contrast, report incidents under a general patient-safety procedure managed by the Medical Administration, which is not specific to medical devices (17). When an incident is identified as involving a medical device, it is forwarded to the Medical Device Division, and some hospital units also report device-related events directly and voluntarily. There is currently no mandatory requirement to designate specific position holders in institutions or companies responsible for collecting, investigating, and transferring adverse event data. While patient-safety training is well established in most healthcare facilities through risk-management units, these programs are broad and not specifically focused on medical-device vigilance. Reporting practices therefore rely largely on institutional reporting routines and high voluntary compliance, guided by Ministry of Health directives and evolving local procedures. The Ministry's well-established pharmacovigilance system for pharmaceuticals illustrates its capacity and commitment to structured post-market safety (18), providing a potential model for advancing medical device vigilance. Building on this experience, efforts to enhance infrastructure for coordination and standardization are ongoing. In the absence of an active law, differing interpretations of reporting expectations may still lead to variation in practice and uncertainty in follow-up and accountability.

Adverse events involving medical devices often differ in nature from other clinical incidents, due to the complex interplay between user error, device malfunction, maintenance procedures, and patient conditions (5, 19–22). Unlike medication-related errors, where causality may be more straightforward, device-related incidents frequently require multifaceted investigation to determine whether the event stemmed from product malfunction, improper use, insufficient training, or contextual clinical factors (23). In ambiguous cases, attributing the event to a device malfunction may become a default response among clinical staff, particularly in the absence of conclusive evidence. On the other hand, manufacturers may tend to view such events as stemming from user-related factors. Moreover, responsibility is often distributed across multiple actors—including clinical staff, biomedical engineers, importers and manufacturers—each with distinct perspectives and roles in the reporting process. This complexity can hinder root cause analysis and delay corrective actions, particularly in the absence of structured communication and shared accountability mechanisms.

While qualitative studies have examined adverse event reporting related to medical devices in various international contexts, they have typically focused on clinical settings and frontline staff, such as physicians and nurses (4–6). In the Israeli context, prior research has addressed adverse events in surgical environments and highlighted factors such as teamwork, psychological safety, and perceptions of “never events” among healthcare professionals (24, 25), but there is no published literature on the factors that influence medical device adverse event reporting in Israel. Additionally, both in Israel and globally, there remains a lack of studies that simultaneously integrate the regulatory, clinical, and industry perspectives within a single analytical framework. Capturing the interplay among these actors is particularly important in environments where formal regulation is still evolving. In this context, it is essential to explore how key stakeholders—healthcare institutions, medical device companies, and the Ministry of Health—experience and navigate the reporting process. Against this backdrop, the objective of this study is to qualitatively identify factors related to medical device adverse event reporting, including stakeholder interests, barriers, perceptions, and attitudes within the Israeli healthcare system.

## 2 Methods

### 2.1 Approach

A reflexive thematic approach (26, 27) was used to explore the perceptions, behaviors, practices, and experiences in relation to the use of medical devices. A total of 31 semi-structured interviews were conducted with stakeholders from three sectors—17 from healthcare institutions, 10 from medical device companies, and 4 policy-makers from the Ministry of Health- representing a diverse range of roles and organizational settings. These interviews aimed to uncover the interests that exist between these groups, as well as the barriers, facilitators, reporting behaviors and inter-organizational relationships related to medical device adverse events. This approach is well suited for analyzing complex organizational and inter-sectoral dynamics, as it allows for the systematic identification of patterns across diverse accounts while remaining attentive to contextual nuance. Thematic analysis involves an iterative process of open coding, constant comparison across transcripts, and the inductive development of broader categories and themes that reflect shared meanings within the data**.** The interview protocols are provided in Supplementary Material 1. Each interview lasted 30–60 min and was conducted between February and December 2024. Three interviews were conducted face-to-face, 25 via Zoom, and three by telephone. All interviews were audio-recorded and later transcribed using the *Transcriptor* software. Ethics approval was obtained from the University of Haifa Ethics Committee (approval no. 350/22), and each participant signed an informed consent form prior to the interview.

### 2.2 Sample selection and recruitment

Participants were purposely selected to ensure a diverse representation of roles within each stakeholder group. In healthcare institutions, interviewees included four physicians, three nurses (including head nurses), four risk-management professionals, three biomedical engineering managers, and one senior administrator, drawn from public hospitals, privately owned hospitals, and hospitals affiliated with health maintenance organizations in various regions across Israel. Two additional participants represented non-hospital settings—a deputy laboratory manager from a laboratory affiliated with a health maintenance organization and a risk-management director from a geriatric care facility. Within medical device companies, participants held a range of positions, including two chief executive officers, four regulatory affairs managers, two application specialists, one procurement manager, and one former director of a commercial representative body. To expose the study to a broad range of device types, both healthcare professionals and company representatives were chosen from various clinical and technological specialties. Interviewees from the Ministry of Health were professionals involved in the oversight and regulation of medical device incident reporting. Participants' demographic characteristics, including sector, gender, and role, are summarized in Table 1. Prospective interviewees were invited by electronic mail. The interview guide was adapted slightly for each sector to ensure relevance while maintaining consistency in the core themes explored. Interviews were conducted until empirical thematic saturation was reached (28), meaning that no new themes emerged in the later stages of data collection.

**Table 1:** Participant demographics by sector, gender, and role.

Sector	Number of participants	Female	Male	Roles/Titles
Ministry of Health	4	2	2	Department Heads from: Patient Safety, Medical Devices Division, Pharmacy Division, Medical Division
Healthcare Institutions	17	9	8	Risk Management Department Manager (5); Head of Department – Physician (1); Physicians (3): Cardiologist, Surgeon, Emergency Room Doctor; Head Nurse – Operating Room (1); Deputy Head Nurse – Delivery Room (1); Nurse (1); Laboratory Director (1); Biomedical Engineering Managers (3); Deputy Hospital Director (1)
Medical Device Companies	10	5	5	CEOs (2); Regulatory Affairs Managers (4); Application Specialists (2); Procurement Manager (1); Former Director of a Commercial Representative Body (1)
**Total**	**31**	**15**	**16**	

### 2.3 Data analysis

The analytical process followed a reflexive thematic analysis (26, 27), combining deductive and inductive approaches (29, 30) to identify patterns and recurring ideas across the interviews. The initial coding and development of the codebook were performed by the first author, who independently coded all transcripts and iteratively refined the codes. To enhance the reliability of the coding framework, a second researcher independently coded a subset of transcripts, and differences were discussed until agreement was reached. Initially, 29 codes were generated deductively from the interview guide, while additional inductive codes emerged during repeated readings of the transcripts, capturing patterns not explicitly addressed in the questions. The analysis was carried out using ATLAS.ti software version 25.0.1.32924 and followed an iterative, comparative process in which codes were refined and grouped into broader categories. Ultimately, the most dominant and relevant codes were consolidated into four overarching themes. The analysis was conducted collaboratively by the research team, who discussed and reached consensus on emerging themes to enhance consistency and resolve differences in interpretation. Thematic saturation, as defined above, was confirmed during the final stage of data analysis. To ensure methodological transparency, the study follows the Consolidated Criteria for Reporting Qualitative Research (COREQ) (31); a completed COREQ checklist is provided in Supplementary Material 2.

## 3 Results

This section presents the main findings from interviews with key stakeholders involved in adverse event reporting related to medical devices in Israel. The analysis yielded four overarching themes:

1. **Intra- and Inter-organizational Communication and Reporting Dynamics**—describes how reporting practices are shaped by informal communication, varied expectations, and the absence of shared procedures across and within institutions, companies, manufacturers, and regulators. This theme encompasses several key interfaces along the reporting chain.
2. **Causality and Responsibility**—examines how stakeholders interpret ambiguous situations and negotiate responsibility when the root cause of an event is uncertain.
3. **Hierarchies and Professional Roles**—examines how professional identity and managerial attitudes influence reporting. This theme includes:
  3.1. *Physicians vs. Nurses*—differences in awareness and engagement with reporting.
  3.2. *Managerial Influence*—the role of leadership in shaping reporting culture.
4. “**What happens with my report?”**—addresses the importance of feedback and follow-up in encouraging reporting.

The following section presents each theme, supported by participant quotes and analysis, illustrating the structural and interpersonal factors that shape reporting practices in the current regulatory environment.

### 3.1 Intra- and inter-organizational communication and reporting dynamics

**Figure 1:**
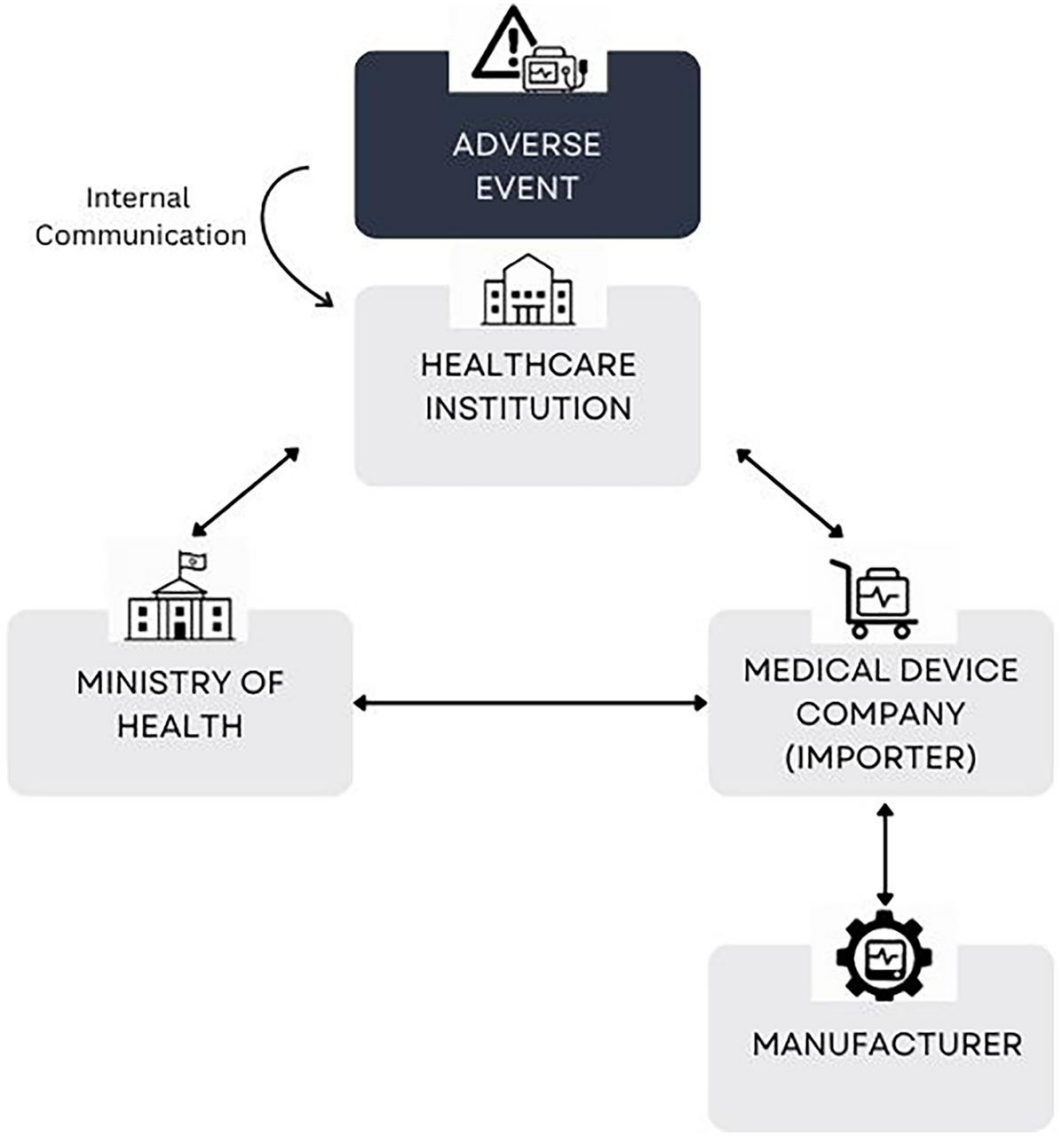
Information and reporting flows between key stakeholders in medical device adverse event reporting.

The reporting of adverse events involving medical devices is shaped not only by internal processes within healthcare institutions, but also by the nature of relationships between key stakeholders: healthcare institutions, medical device companies (including importers and manufacturers), and regulatory authorities. The findings suggest that responsibilities and communication channels—particularly between healthcare institutions and medical device companies—are not always clearly defined or standardized. Cooperation was often described as informal and case-dependent. Participants also noted a perceived lack of enforcement mechanisms and regulatory clarity, which, in some cases, contributed to inconsistent reporting practices. The following findings are presented in accordance with key communication dynamics identified between the main actors involved in adverse event reporting. These relationships are illustrated in Figure 1, which maps the flow of information and interaction across institutions.

#### 3.1.1 Communication between healthcare institutions and medical device companies

Interviewees from medical device companies described inconsistent and sometimes insufficient information transfer from healthcare institutions following adverse events. While they expressed frustration over receiving fragmented or unofficial reports, they also acknowledged that the level of cooperation often depends on the specific case and the individuals involved. In some instances, they reported excellent collaboration and timely access to the necessary data. Similarly, certain healthcare professionals believed they were providing all relevant information. This variation suggests a misalignment of expectations and a lack of shared protocols, which may hinder the ability to conduct thorough investigations and improve safety outcomes.

A regulatory affairs manager at a medical device company explained: “*If I want to provide the manufacturer with information about a device issue so they can investigate it properly, I need all the data, the full story. I know hospitals have this information, but we don't receive it. Sometimes they even discard the product, so the manufacturer has nothing to examine.*”

Similarly, an applications manager described the lack of formal processes: “*We rarely receive a formal report. Usually, someone casually mentions a question, and I have to guess that an incident occurred. There's no protocol, no consistent contact point—sometimes it's the department, sometimes engineering, sometimes a technician. I can't transfer the information properly this way.*”

From the perspective of a deputy hospital director, the issue was framed differently: “*I believe we share all the information we can. There's no reason for us to withhold anything, unless it's just due to workload or the brevity of communication. But in general, I think we're professional in our work.*”

A representative from the Ministry of Health described a recurring pattern in which hospitals report adverse events both to the Ministry and to companies, yet often provide companies with only partial or minimal information. She noted that reluctance to engage openly with companies may stem from mistrust or strained relationships, occasionally requiring the Ministry's mediation.

This theme suggests a misalignment in perceptions and practices surrounding information sharing between healthcare institutions and companies. While companies feel they lack access to critical data, healthcare institutions may not fully recognize the implications of these gaps or the companies' investigative needs. Such differences in perception and practice may, in some cases, hinder companies' ability to fully investigate adverse events and support prevention efforts.

In response to these communication gaps, some medical device companies have developed internal strategies to detect adverse events, even in the absence of formal reports. These include proactive approaches that train employees to recognize indirect signals—such as casual remarks or seemingly unrelated questions—that may indicate a product issue.

As one regulatory manager explained: “*We emphasize what we call ‘a trained ear'—an adverse event doesn't always come as an adverse event. It may appear as a story or a question. We monitor these cues and translate them into our internal language. Instead of using ‘adverse event,’ we speak in terms people relate to, like ‘complaint.’ Then we assess whether it meets the reporting criteria.*”

This interpretive skillset reflects companies' reliance on informal communication, interpersonal awareness, and internal analysis as substitutes for consistent, structured reports. This adaptive behavior reflects companies' efforts to bridge communication gaps that occasionally arise and compensate for the lack of structured reporting from healthcare institutions.

#### 3.1.2 Internal communication within healthcare institutions

According to interviewees from medical device companies, there appear to be internal communication breakdowns within healthcare institutions, where information may be lost or distorted as it moves through the reporting chain. While they described some cases in which hospitals provided clear and timely documentation, they also recounted instances where the information flow was fragmented, and those reporting the incident were not the ones who directly experienced it. This, in their view, led to partial or inaccurate accounts, making it challenging to identify the equipment involved or trace key details, particularly when the initial data is not properly recorded or shared.

As one product manager in a company explains: “*It's very difficult to understand exactly what happened, what the team did, and which equipment was used. Sometimes even identifying the device is hard. By the time we find the person involved, they no longer remember the details, and there's usually no formal report.*”

Similarly, an application specialist highlighted the fragmented nature of communication: “*We often get inquiries from someone who's the third or fourth link in the chain. The distortion grows with each step, and often they don't know who reported it first. Eventually, the information is incomplete, and they can't move forward because the details are missing and the issue remains unclear.*”

These accounts underscore potential communication gaps and suggest a need for clearer internal protocols and more systematic documentation to support accurate and timely event tracking.

#### 3.1.3 Communication between healthcare institutions and the ministry of health

Healthcare institutions follow the Ministry of Health's guidelines, and the reporting process is generally perceived as clear. However, this reporting is largely based on trust, as there is no enforcement mechanism in place. Institutions value the Ministry's involvement, as it helps mediate between them and the companies, though they may not always be eager to share full details. Furthermore, reports about medical equipment-related adverse events often reach multiple parties, creating inefficiency and confusion due to an unclear reporting chain.

A risk management manager at a healthcare institution explains: “*There's a clear directive from the Ministry of Health with defined criteria for reporting, which is very clear to us. In the case of an adverse event involving medical device, we prefer that the Ministry of Health contacts the companies directly, as they have more leverage over them. The companies don't want to be contacted by the Ministry of Health*.”

A representative from the Ministry of Health also supported this view, noting that hospitals often perceive the Ministry's involvement as increasing the manufacturer's responsiveness.

Shifting focus to communication-related inefficiencies involving multiple parties within and outside the hospital, one medical engineering manager at a healthcare institution described a situation in which he might receive up to ten identical emails regarding the same issue in a single day, often having to provide the same response repeatedly—a situation that, in his view, reflects the lack of clear reporting responsibilities and contributes to inefficiencies in communication.

Legal concerns may also affect the willingness of hospitals to share information. A reporting manager at the Ministry of Health explained: “*When an incident occurs, hospitals often keep information to themselves. It's part of defensive medicine. If there's concern about legal action, they become even more guarded.*”

Building on this perspective, the same Ministry of Health representative further noted that while cooperation with hospitals does occur, it is often unstructured and constrained. In the absence of a regulatory framework, the Ministry's ability to engage hospitals may be limited—particularly in cases involving patient harm, where hospitals, as the primary affected parties, may be more cautious in sharing information due to concerns about potential legal consequences.

This subtheme highlights both alignment and tension in the relationship between healthcare institutions and the Ministry of Health: while the reporting process is perceived as clear and the Ministry is viewed as a valuable intermediary, challenges remain due to trust-based reporting, legal sensitivities, and inefficiencies such as duplicated communication.

#### 3.1.4 Communication between medical device companies and the ministry of health

Although the Medical Devices Law is not yet in effect and no formal reporting obligation currently exists, several company representatives—particularly from larger firms—demonstrated a strong awareness and commitment to reporting adverse events. This tendency appears to be reinforced by contractual obligations to manufacturers, who often require systematic documentation and communication. The interaction with the Ministry of Health is generally described as cooperative, though the absence of enforceable regulation leaves some regulators feeling limited in their authority.

The following quotes illustrate the industry's proactive stance, particularly in contexts where reporting aligns with manufacturer expectations or internal company norms: A regulatory affairs manager at a medical device company explained:

“We report everything. There are no conflicting interests. If there's doubt—there's no doubt. Especially if the manufacturer says to report, then it's absolutely clear. Everything is well-organized.”

According to a former director of a commercial representative body that liaises regularly with medical device companies, there is broad agreement within the industry regarding the importance of reporting. She noted that when reporting is required—either by the manufacturer or due to deviations from expected device performance or from standard instructions—it is done without hesitation.

A senior manager involved in incident reporting at the Ministry of Health described these interactions in positive terms: “*Most of the time, there's very good and high-level cooperation. It's quite amazing, really. Even though there's no official regulation and the guidelines are just a draft—and not widely disseminated—the regulatory staff at the companies still choose to report and collaborate quickly and positively.*”

At the same time, the lack of legal enforcement tools remains a concern. As another Ministry of Health manager involved in incident reporting explained: “*It's frustrating that sometimes we want to apply sanctions against a manufacturer, but since the law isn't in effect, we have no authority to act. The regulator needs more power—more teeth—when it comes to both manufacturers and suppliers. Right now, we're powerless.*”

These accounts suggest that, despite the absence of binding regulation, cooperation between companies and the Ministry of Health is often strong. This collaboration appears to be supported by internal company practices and by contractual obligations to manufacturers, which encourage systematic reporting.

#### 3.1.5 Communication between medical device companies and the manufacturer

This subtheme focuses on communication between Israeli medical device importers and the global manufacturers they represent. Formal contracts require importers to report adverse events and follow manufacturers' instructions. At the same time, importers often find themselves balancing these obligations with the need to maintain trust and responsiveness toward local healthcare providers. This dual responsibility can create tensions when expectations diverge.

This dynamic is illustrated in the following accounts, which reflect how importers navigate these conflicting demands in practice. One regulatory affairs manager explained:

“There's no discretion when it comes to what we report. We have to forward everything the manufacturer sends us. Ultimately, the manufacturer is responsible for the product and risk management—we trust their lead.”

An employee in a procurement role described a case where tensions arose between a manufacturer's investigative process and the healthcare provider's expectations:

“Sometimes we report a malfunction to the manufacturer and they begin asking follow-up questions—sometimes too detailed. I recall one case where the doctor eventually said, ‘That's enough, it malfunctioned, I've said all I can.’ The hospital didn't want to continue the back-and-forth. The manufacturer insisted the product was fine, but the hospital said, ‘We don't want it anymore, we no longer consider it reliable.’ In the end, our role is to support the hospital while also fulfilling our obligation to the manufacturer.”

These examples illustrate how communication with manufacturers is shaped by a sense of procedural obligation, while simultaneously requiring importers to navigate sensitivities and expectations within the local clinical environment, including the need to preserve relationships and reputational trust with clinical clients.

### 3.2 Who's to blame? complexities of causality and responsibility in medical device incidents

**Figure 2:**
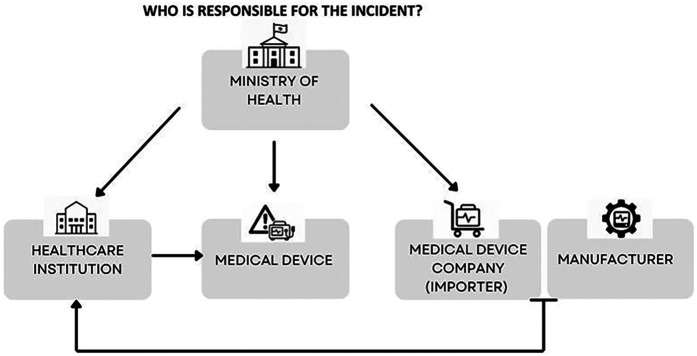
Dynamics of responsibility attribution in medical device incidents when the cause is unclear. Responsibility tends to shift between the medical staff and the medical device (as represented by the manufacturer).

Adverse events involving medical devices can arise from a variety of factors, including technical malfunction, improper use, inadequate maintenance, or the patient's clinical condition. Given this complexity, each event typically prompts an investigation aimed at identifying the root cause to ensure accountability and to prevent recurrence. However, identifying the incident cause is not always straightforward. The device itself may become an additional actor in the event, alongside the medical team and the patient. When the cause is unclear, various parties may seek to distance themselves from responsibility—healthcare professionals may attribute the problem to a device malfunction, while manufacturers, who invest heavily in designing reliable equipment, often emphasize issues related to maintenance or use. This tendency to shift blame when causality is ambiguous is reflected in the dynamics described in Figure 2.

The tendency to shift the blame was mentioned by interviewees from different sectors. For example, a head nurse in an operating room noted that both hospital staff and companies tend to deflect blame:

“The Company will say it's not their fault—just like I say it's not me, it's the device. In Israel, the norm is to shift responsibility. Everyone passes it along.”

A CEO of a medical device company echoed this dynamic from the perspective of a medical device company, suggesting that hospitals may hesitate to report incidents when the fault lies in misuse: “*Hospitals often avoid reporting when there's a user error—incorrect programming, improper use, or other mistakes. There's an incentive to blame the product rather than admit fault. But when the issue is clearly product-related, there's no conflict of interest*.”

Regulators also encounter this blame game. A manager at the Ministry of Health involved in incident reporting noted: “*If you ask the manufacturers, it's always the medical staff's fault. Cases where a company actually admits to a product issue—you can count those on two hands. In my experience, I know what the answer will be before I even ask: ‘There's no problem with the device; the problem is with the staff who didn't use it properly.’ And I don't agree with that*.”

Another manager from the Ministry of Health reinforced this depiction of the blame game, noting that healthcare institutions typically assert their staff used the device correctly and place the blame on the equipment. He added that healthcare institutions tend to be less forthcoming when follow-up questions are asked.

At the same time, some interviewees from within hospitals emphasized that human factors are indeed the most common contributors to such events. One deputy hospital director estimated:

“It's the 80/20 rule—80% of events are due to human error: improper use, failure to check equipment, use outside indications. Only about 20% are related to equipment failure or mismatch.”

In summary, some interviewees described instances in which responsibility for an adverse event was shifted back and forth between clinical staff and medical device manufacturers. At the same time, several stakeholders from healthcare institutions supported the view that most adverse events involving medical devices are primarily related to human factors.

### 3.3 Hierarchies and professional roles

The organizational and professional hierarchies embedded within healthcare institutions shape the ways in which adverse events are reported and managed. Participants described how professional identity, perceived authority, and role expectations influence both the willingness to report and the experience of reporting. In particular, two subtopics emerged within this theme: Physicians vs. Nurses: Cultural and Behavioral Gaps in Reporting—reflecting distinct professional cultures, levels of awareness, and behavioral norms related to adverse event reporting; and The Role of Managerial Attitudes in Fostering a Reporting Culture—emphasizing how leadership can promote psychological safety and continuous learning.

#### 3.3.1 Physicians vs. nurses: cultural and behavioral gaps in reporting

Interviews revealed notable disparities in reporting practices and awareness of adverse event protocols between different professional groups, particularly between physicians and nurses. These differences are linked to varying levels of familiarity with reporting procedures, professional culture, and implicit divisions of responsibility. A recurring theme was the implicit expectation that nurses would take the lead in reporting, while physicians often assumed a more peripheral role. The following accounts demonstrate how these professional dynamics play out in everyday reporting practices, revealing gaps in responsibility and knowledge.

As one risk management officer explained: “*This is a global phenomenon. In our case, nurses report about 97% of the events. It's mainly a matter of safety culture and perception. Nurses understand that reporting is about preventing the next event. They are closer to the patient, and they grasp the importance of reducing risks. In terms of a no-blame approach and understanding the value of reporting—they are two steps ahead of everyone else. Physicians report significantly less, and that's a real challenge.*”

This implicit division of responsibility sometimes creates tension between the professions. One operating room head nurse put it this way: “*Only nurses report. Only nurses! Doctors don't report anything. Anesthesiologists don't report anything. Nobody reports. But in the end, I'm the one who takes the heat. Nurses always take the fall. Always.*”

One nurse described how the need to preserve collegial relationships with senior physicians could create tension in real-time decision-making around reporting. In some cases, raising concerns was perceived as potentially disruptive to future collaboration and workplace harmony.

One physician acknowledged that nurses tend to be more familiar with protocols and more engaged in the reporting process: “*I get the impression that the nurses are more responsible for this. They work by clear protocols and are very thorough. Even though we're all obligated to report, I think they take more initiative.*”

Discrepancies in knowledge and awareness of the reporting process were also evident, as reflected in the following contrasting accounts by a physician and a nurse. One physician admitted: “*I didn't need to report, so I didn't look into it. I never encountered it and didn't participate in any training sessions about it.*” Conversely, one nurse described a highly structured approach to reporting within her unit, including formal documentation and follow-up meetings with risk management.

These accounts underscore how professional culture and informal hierarchies shape reporting behavior. Nurses were often seen as bearing greater responsibility for reporting, due to expectations and procedural familiarity. This perceived imbalance may reflect unclear role divisions and, at times, lead to friction between nurses and physicians.

#### 3.3.2 The role of managerial attitudes in fostering a reporting culture

Another critical dimension within the broader theme of professional hierarchies is the influence of managerial attitudes—commonly referred to by participants as “the tone set by the manager”—on the willingness of staff to report adverse events. Interviewees emphasized that the immediate reaction of direct supervisors plays a key role in shaping staff perceptions regarding the consequences of reporting. Fear of blame or punitive responses emerged as a strong deterrent.

A senior risk management officer at a large hospital noted: “*We realized that what shapes employees' fear of repercussions isn't some abstract system—it's their direct manager. The first reaction they get from their manager after they report an incident is what really matters. Sometimes, even a comment like ‘I can't believe you did that' is enough to make someone shut down.*”

This sentiment was echoed by frontline staff, who described how negative managerial reactions can undermine trust and discourage further disclosure. One nurse shared a personal experience of being harshly reprimanded over the phone by her department head shortly after completing a night shift. The manager did not ask for any context or explanation, and instead responded with anger. The nurse described feeling deeply shaken by the encounter and later confronted the manager, saying that such a reaction deters staff from reporting, as it conveys blame and makes them feel personally responsible for the outcome—as though they were being held accountable for the entire incident.

In contrast, several participants emphasized that when managers demonstrate empathy and avoid assigning personal blame, staff feel safer to report. A deputy head nurse reflected: “*When the person in charge of risk management takes a non-blaming approach, many more nurses feel comfortable reporting.*”

This view was reinforced by another risk management leader, who described an intentional cultural shift: “*You can give endless lectures about how important reporting is, but if someone reports and gets scolded—‘Why did this happen?’—then we've lost them. That's why we never allow blame in these discussions. We focus on system-level learning, not personal fault.*”

Similarly, a biomedical engineering manager observed that when leadership encourages reporting, staff tend to see it as part of their role—but when such support is lacking, motivation declines.

Taken together, these accounts underscore how managerial tone—whether supportive or punitive—shapes not only individual decisions to report, but also the broader organizational culture surrounding safety and accountability.

### 3.4 “What happens with my report?”

A recurring theme across interviews from all sectors—healthcare institutions, medical device companies, and the Ministry of Health—was the recognition of the importance of feedback and visible outcomes following the submission of adverse event reports, as well as the negative impact of their absence. Participants emphasized that knowing what happens with a report—whether it leads to action or acknowledgment—plays a critical role in encouraging continued reporting.

Within healthcare institutions, both risk managers and biomedical engineering staff emphasized that adverse event reports can serve as a meaningful tool for promoting change. When reports lead to visible outcomes—such as equipment replacement, discussions in reuse committees, or official responses—staff feel that reporting is worthwhile and are more likely to continue doing so. As one risk manager put it: “*If the staff doesn't see what is done with the reports, they may become less inclined to report. When they see seriousness and actions taken, they report more.*”

A few participants, primarily from healthcare institutions, noted that they do not always receive feedback from the Ministry of Health. As one hospital risk manager explained, this lack of response makes it difficult to know whether reports were reviewed or led to any action: “*It's not really clear what they [the MoH] do with our reports. They respond to a few, but it's a very small portion*.” Similarly, a biomedical engineering manager said he could not recall ever receiving feedback.

From the perspective of industry, a company representative emphasized the value of understanding how a report fits into a broader context: “*I'd want someone to tell me: you submitted a report, and you're one of 800 who did the same worldwide. That kind of feedback makes it a two-way street.*”

In response, a representative from the Ministry of Health acknowledged the challenge of providing feedback to healthcare institutions, citing limited resources. She explained that not every report leads to regulatory action, and that staffing constraints make it difficult to respond to each report individually: “*If we could respond to each report, thank the staff member, and explain what we did—that would boost motivation. But we just don't have the personnel.*”

Some interviewees from healthcare institutions noted that, in contrast to the Ministry of Health, medical device companies were often more responsive to adverse event reports. This responsiveness was perceived as a sign of seriousness and engagement on the part of the companies. As one hospital risk manager observed: “*They [the device company] take it seriously. When I contact them, I almost always get a response—like, ‘we're coming in two hours to collect the product.’ Or they'll say, ‘this is the third event in four months and it's under global investigation.’ I can't say the same about the MoH.*”

Taken together, these accounts suggest that timely, transparent, and meaningful feedback—whether from regulators, companies, or from within the healthcare institution (e.g., visible actions taken in response to reports)—plays an important role in how reporting is perceived and sustained over time.

## 4 Discussion

This study explored the perceptions, behaviors, and communication patterns among key stakeholders involved in reporting adverse events related to medical devices in Israel, including healthcare institutions, medical device companies, and the Ministry of Health. The findings highlight complex dynamics of responsibility attribution, communication challenges, and the influence of organizational culture on reporting behavior. While some of the themes that emerged—such as inter-organizational communication and the shifting of blame—are closely tied to the unique characteristics of medical devices, others—such as role hierarchies and the impact of feedback—reflect broader challenges common to adverse event reporting across healthcare contexts (32, 33). These findings shed light on the interplay between formal systems and informal practices in a regulatory environment that is still in development (32, 34).

### 4.1 Communication barriers and fragmented information flow

The findings indicate that communication processes among stakeholders are often fragmented and lack formal structure. The absence of clear reporting protocols and regulatory mandates contributes to inconsistent information flow, particularly between healthcare institutions and medical device companies. While some healthcare professionals perceive manufacturers as responsive and proactive in addressing adverse events, for example by initiating investigations or improving devices, the relationship is also shaped—according to prior research—by underlying interests such as ongoing contracts, dependence on training and implementation support, and the need to maintain productive ties (6). Concerns about potential legal repercussions may further inhibit transparency (35). In addition, local importers often serve as intermediaries between clinical settings and global manufacturers. This dual role places importers between two loyalties: complying with international regulatory expectations while maintaining local clinical relationships and trust. While collaboration with medical device companies was generally described as positive, some company representatives acknowledged concerns about potential reputational damage, financial loss, or negative impact on product marketing. Although these concerns were not commonly cited, they may contribute to communication hesitancy in certain cases of adverse event reporting.

Within healthcare institutions, internal communication breakdowns—where information passes through multiple intermediaries—may lead to distorted or second-hand accounts that undermine the accuracy of reports. These gaps highlight the absence of standardized procedures for immediate documentation and clear role definitions. A similar concern was raised in a previous study, which found that key identifying information about the device used is often missing from patient records, hindering traceability in the event of a later investigation (5). In parallel, some interviewees from healthcare institutions described excessive and duplicative communication regarding the same incident, reflecting a lack of centralized responsibility and contributing to inefficiencies in inter-organizational information flow. These communication failures mirror challenges commonly reported in complex healthcare settings (36). The lack of standardization also affects interactions with external entities, including regulatory authorities.

Communication between healthcare institutions and the Ministry of Health appears to be more structured, with institutional actors expressing trust in the regulator's role and authority. Some healthcare representatives prefer that the Ministry of Health act as an intermediary when engaging with manufacturers, believing that regulatory involvement may prompt more thorough responses. However, this preference also reflects a lack of direct accountability and a diffusion of responsibility among stakeholders. In parallel, concerns about potential legal liability may further limit the willingness of healthcare institutions to share detailed information with the Ministry, especially when legal consequences are feared (35, 37). These communication gaps not only limit the ability to investigate adverse events effectively, but also create fertile ground for ambiguity and deflection of responsibility. Stakeholders may be more inclined to distance themselves from fault—intentionally or otherwise—especially in the absence of structured coordination or regulatory expectations. The next section explores how such dynamics of blame attribution unfold among healthcare institutions, medical device companies, and regulators.

### 4.2 Ambiguity of responsibility and the “Blame Shift” phenomenon

The findings reveal that ambiguity surrounding the cause of device-related incidents often leads to shifting responsibility among stakeholders. While extensive research has examined errors within a systemic framework—focusing on identifying their causes (38, 39)—the present study emphasizes the dynamics of blame and accountability that unfold primarily between healthcare providers and medical device companies, while also reflecting broader systemic interactions involving the Ministry of Health. When causality is unclear (22), each party tends to attribute fault to others: healthcare institutions may point to technical malfunction, whereas manufacturers often emphasize user error or improper maintenance. This dynamic is frequently shaped by concerns over legal liability, professional reputation, or commercial interests—particularly among manufacturers seeking to protect their product's image. A representative from the Ministry of Health expressed frustration with what she described as a predictable pattern in manufacturers' responses: systematically attributing incidents to user error, regardless of context. Such perceptions erode trust and highlight the need for balanced and transparent investigation processes. Healthcare institutions, too, may hesitate to report events involving staff error due to fear of litigation or internal disciplinary consequences. However, a quantitative study from England found an inverse association between reporting rates and malpractice claims: hospitals with higher levels of patient safety incident reporting tended to experience fewer legal claims (40). This finding suggests that transparent reporting may actually reduce legal exposure, rather than increase it.

These dynamics hinder open communication and obstruct efforts to identify root causes and implement preventive measures. The involvement of multiple actors—hospitals, importers, manufacturers, and the Ministry of Health—without clearly defined processes for responsibility-sharing or incident analysis further complicates resolution. Although similar challenges have been observed even in regulated settings (2), the absence of a formal framework may heighten these tendencies. These findings highlight the need for more coordinated practices and shared understanding across stakeholders to support learning from adverse events. They also echo broader patterns described in the literature, where unclear causality, reputational concerns, and fear of liability have been shown to contribute to reluctance in accepting accountability and to systemic blame deflection (41, 42).

### 4.3 Cultural and organizational influences on reporting behavior

Professional hierarchies and organizational culture emerged as central factors shaping reporting practices. This finding aligns with previous qualitative research on adverse event reporting in clinical settings, which found that physicians often do not perceive reporting as part of their role to the same extent as nurses (33, 35). In our study, nurses were described as more active reporters, often viewing the act of reporting as part of their responsibility to patient safety. In contrast, physicians were reported to engage less frequently in reporting, sometimes due to unfamiliarity with procedures or social dynamics that discourage admission of error. One nurse described a sense that reporting responsibilities were disproportionately placed on nurses, even when physicians were equally involved. This perceived imbalance led to concerns about fairness, professional boundaries, and potential strain on collegial relationships. In situations where nurses believed reporting was necessary but physicians disagreed, the desire to preserve collegial relationships sometimes led to reluctance, highlighting the complex interpersonal dynamics, as also noted in prior research highlighting mismatched expectations between nurses and physicians (6). These dynamics are consistent with prior studies demonstrating that professional hierarchies, perceived roles, and a lack of psychological safety can shape reporting behaviors in clinical settings (43–45). Addressing disparities in awareness, perceived responsibility, and inter-professional expectations may be essential for fostering a more equitable and effective reporting environment. These gaps may also reflect the absence of a formal nationwide training program on device vigilance, which contributes to uneven familiarity with reporting procedures and responsibilities.

Managerial attitudes were also found to play a decisive role in shaping the willingness to report. Supportive, non-punitive responses from supervisors were associated with higher levels of engagement, while fear of criticism or disciplinary action deterred staff from reporting. Similar findings have been reported in previous studies (40, 46). These findings align with broader understandings of the importance of psychological safety in healthcare settings and suggest that leadership development may be a key component of improving reporting culture (47, 48).

### 4.4 The role of feedback in sustaining motivation

Across all sectors, participants emphasized the importance of feedback following report submission—whether from hospital management to clinical staff, from regulators to hospitals, or from regulators to companies. The perception that reports “disappear” or fail to generate action was associated with frustration and reduced motivation to report. Conversely, instances in which reports led to equipment replacement, process improvement, or acknowledgment from regulators or companies were cited as positive reinforcement. These findings suggest that feedback mechanisms are not only a matter of transparency but also a critical driver of engagement. Similar concerns have been noted in Australia, where limited regulatory follow-up on adverse event reports has raised questions about system responsiveness (49). The importance of visible and timely responses is well-documented, as the absence of feedback is associated with underreporting and disengagement (6, 50). Notably, one study also indicates that healthcare professionals may perceive manufacturers as more responsive than other stakeholders (6)—a perception echoed in this study. However, ensuring consistent feedback requires time, personnel, and institutional support—resources that are often limited.

Recent international studies reinforce the relevance of these findings and provide comparative perspectives that inform the policy directions proposed later in this paper. Evidence from multiple health systems indicates that the effectiveness of post-market surveillance depends on both the existence of clear and enforceable regulatory frameworks and their consistent implementation (51). Transparent collaboration and communication among stakeholders are equally vital, as close relationships between healthcare institutions and manufacturers may influence decision-making and accountability (52, 53). Recent research further emphasizes that clear reporting protocols, harmonized terminology, and continued education are essential to ensure consistent understanding and effective communication across sectors (54). Building on the finding of the present study that more immediate documentation within hospitals is needed, recent analyses show that real-time digital reporting and comprehensive data exchange between healthcare institutions and manufacturers can further enable accurate post-market evaluations and regulatory compliance (55). Hospital-based vigilance systems similarly demonstrate that structured digital systems for real-time event recording can improve reporting rates and strengthen overall monitoring capacity (56). Moreover, research on inter-professional collaboration shows that constructive physician–nurse relationships are closely linked to patient safety culture, underscoring the importance of defining complementary reporting roles that foster collaboration across professional boundaries (57). Evidence from communication interventions demonstrates that fostering psychological safety within healthcare teams can enhance open dialogue and strengthen patient-safety culture (58, 59). A scoping review of nurses' experiences with incident reporting highlights that supportive managerial attitudes, regular feedback, and targeted training can strengthen motivation to report and foster a culture of openness, trust, and shared responsibility (60). Similarly, a recent study highlights that inter-organizational collaboration—grounded in effective communication—can enhance coordination and system efficiency, reinforcing the present study's findings on the importance of trust and collaboration among regulators, healthcare institutions, and medical device companies (61). Together, these insights provide an international context that underpins the policy recommendations formulated in this study.

### 4.5 Limitations and future research directions

While the discussion highlights several factors shaping reporting practices, the interpretation of these findings must be considered in light of certain study limitations. Although efforts were made to include a diverse range of participants across sectors and professional roles, the sample may not fully represent all perspectives involved in medical device adverse event reporting. For example, the fact that most company representatives in this study described proactive reporting practices does not necessarily indicate that all companies behave similarly; it is possible that firms not represented in the sample may be less engaged in reporting. In addition, some degree of selection bias may have occurred, as participation was voluntary and those who agreed to be interviewed might have been more interested or positively inclined toward the topic. The study also relied on a single data source—semi-structured interviews—without additional triangulation through document analysis or observation, which limits the ability to cross-validate participants' accounts. Furthermore, the possibility of social desirability bias cannot be excluded; given the sensitivity of the topic, some interviewees may have provided responses that portray their organization or profession in a favorable light. As a qualitative study, the findings offer in-depth insights into stakeholder perceptions and contextual dynamics but cannot provide frequencies or generalizable prevalence of specific views or framing patterns. However, qualitative research is particularly valuable for capturing the complexity of local practices and the meanings assigned to them by participants (62). Future research could build on these findings by employing mixed methods, including quantitative approaches to assess the prevalence and impact of factors identified in this study—such as communication, responsibilities, differences between physicians and nurses, and the role of feedback. Additional studies could explore how organizational and regulatory changes shape reporting behaviors over time, complemented by comparative analyses across countries.

### 4.6 Conclusions

Although conducted in Israel, this study offers insights with broader relevance to medical device vigilance worldwide. Its main contribution to the literature lies in moving beyond the prevailing emphasis in existing research on healthcare staff by incorporating the perspectives of diverse stakeholders—clinicians, biomedical engineers, and administrators from healthcare institutions, alongside regulators and professionals in varied roles within medical device companies. In the field of medical devices, such multidisciplinary engagement is particularly critical: only by combining clinical, engineering, managerial, and regulatory expertise can adverse events be thoroughly investigated, root causes identified, and future incidents prevented. Viewed as a case study, Israel illustrates how a small and interconnected health system can shed light on dynamics likely to recur elsewhere. Beyond regulatory frameworks and clear role definitions, effective vigilance also depends on transparent communication and trust between healthcare institutions, companies, and regulators. Strengthening these relationships offers lessons for harmonizing reporting practices, enhancing stakeholder engagement, and ultimately improving patient safety worldwide.

#### 4.6.1 Policy implications

- Ensuring that regulatory frameworks are fully implemented and enforced helps to establish clearer expectations and responsibilities among stakeholders.
- At the system level, standardized reporting protocols—clarifying who should report, how information should be transferred, and how devices should be managed post-incident—can reduce ambiguity and enhance the consistency and completeness of reports.
- Regular public reporting by regulatory authorities, summarizing key findings, trends, and actions taken, can reinforce the value of reporting, strengthen stakeholder trust, and promote sustained engagement. These efforts require adequate resources, as meaningful feedback and timely follow-up depend heavily on human resources, digital infrastructure, and analytical capacity.
- Clear delineation of reporting roles within healthcare institutions—for example, between nurses and physicians—may support more coordinated and effective practices.
- At the institutional level, structured internal protocols for documenting adverse events at the point of occurrence—including essential details such as the device's serial number and a clear description of the circumstances—can reduce reliance on informal communication and improve the accuracy and completeness of reports submitted for investigation.
- Strengthened feedback loops across levels of the system, ensuring timely and meaningful follow-up, and encouraging collaboration rather than defensiveness can foster transparency, trust, and shared responsibility.
- Supporting staff through training and clear procedures can further contribute to building a more responsive reporting culture.

## Data Availability

The raw data supporting the conclusions of this article will be made available by the authors, without undue reservation.

## References

[1] Hinrichs-Krapels S Ditewig B Boulding H Chalkidou A Erskine J Shokraneh F. Purchasing high-cost medical devices and equipment in hospitals: a systematic review. BMJ Open. (2022) 12(9):e057516. 10.1136/bmjopen-2021-057516 36581959 PMC9438058

[2] Craig A Meley PO Carter P. The need for greater reporting of medical device incidents. Eur Med J. (2019) 3(1):56–63. 10.33590/emjinnov/10312553

[3] Resnic FS Normand S-LT. Postmarketing surveillance of medical devices—filling in the gaps. N Engl J Med. (2012) 366(10):875–7. 10.1056/NEJMp1114865 22332950

[4] Polisena J Gagliardi A Urbach D Clifford T Fiander M. Factors that influence the recognition, reporting and resolution of incidents related to medical devices and other healthcare technologies: a systematic review. Syst Rev. (2015) 4(1):37. 10.1186/s13643-015-0028-0 25875375 PMC4384231

[5] Gagliardi AR Ducey A Lehoux P Turgeon T Ross S Trbovich P Factors influencing the reporting of adverse medical device events : qualitative interviews with physicians about higher risk implantable devices. BMJ Qual Saf. (2018) 27:190–8. 10.1136/bmjqs-2017-006481 28768712 PMC5867432

[6] Polisena J Gagliardi A Clifford T. How can we improve the recognition, reporting and resolution of medical device-related incidents in hospitals? A qualitative study of physicians and registered nurses. BMC Health Serv Res. (2015) 15(1):220. 10.1186/s12913-015-0886-0 26043923 PMC4456786

[7] Hewitt TA Chreim S. Fix and forget or fix and report: a qualitative study of tensions at the front line of incident reporting. BMJ Qual Saf. (2015) 24(5):303–10. 10.1136/bmjqs-2014-003279 25749025 PMC4413736

[8] Desveaux L Gagliardi AR. Comparing the application of two theoretical frameworks to describe determinants of adverse medical device event reporting: secondary analysis of qualitative interview data. BMC Health Serv Res. (2018) 18:1–14. 10.1186/s12913-018-3251-2 29866152 PMC5987566

[9] European Union. Regulation (EU) 2017/745 of the European Parliament and of the Council. (2017).

[10] McDermott O Kearney B. A review of the literature on the new European medical device regulations requirements for increased clinical evaluation. Int J Pharm Healthc Mark. (2024) 19(1):1–21. 10.1108/IJPHM-07-2023-0060

[11] Mishali M Sheffer N Mishali O Negev M. Evaluation of reporting trends in the MAUDE database: 1991 to 2022. Digit Health. (2025) 11:20552076251314094. 10.1177/20552076251314094 39850626 PMC11755539

[12] FDA. MAUDE—Manufacturer and User Facility Device Experience. Available online at: https://www.accessdata.fda.gov/scripts/cdrh/cfdocs/cfmaude/search.cfm (Accessed November 30, 2025).

[13] Tau N Shepshelovich D. Assessment of data sources that support US food and drug administration medical devices safety communications. JAMA Intern Med. (2020) 180(11):1420–6. 10.1001/jamainternmed.2020.3514 32986074 PMC7522775

[14] Pane J Verhamme KMC Villegas D Gamez L Rebollo I Sturkenboom MCJM. Challenges associated with the safety signal detection process for medical devices. Med Devices Evid Res. (2021) 14:43–57. 10.2147/MDER.S278868 PMC7917351 33658868

[15] Everhart AO Karaca-Mandic P Redberg RF Ross JS Dhruva SS. Late adverse event reporting from medical device manufacturers to the US food and drug administration: cross sectional study. Br Med J. (2025) 388. 10.1136/bmj-2024-081518 PMC11898541 40081838

[16] Ministry of Health. Medical Device Act. Jerusalem: Ministry of Health, Isreal (2012). Available online at: https://www.health.gov.il/LegislationLibrary/Briut50.pdf (Accessed July 21, 2024).

[17] Ministry of Health. Medical Administration Circular No. 2/2021: Mandatory Reporting of Adverse or Unusual Events by Healthcare Institutions. (2021). Available online at: https://www.gov.il/he/pages/mr02-2021 (Accessed April 13, 2021).

[18] Schwartzberg E Berkovitch M Dil Nahlieli D Nathan J Gorelik E. Pharmacovigilance in Israel—tools, processes, and actions. Isr J Health Policy Res. (2017) 6(29):1–9. 10.1186/s13584-017-0154-3 28760141 PMC5537943

[19] Mishali M Sheffer N Mishali O Negev M. Understanding variation among medical device reporting sources: a study of the MAUDE database. Clin Ther. (2025) 47(1):76–81. 10.1016/j.clinthera.2024.10.004 39516116

[20] White GG Weick-Brady MD Gross TP. Improving patient care by reporting problems with medical devices. J Toxicol Clin Toxicol. (1998) 36(6):641–4. 10.3109/15563659809028066 9776975

[21] Knisely BM Levine C Kharod KC Vaughn-Cooke M. An analysis of FDA adverse event reporting data for trends in medical device use error. Proc Int Symp Hum Factors Ergon Health Care. (2020) 9(1):130–4. 10.1177/2327857920091024

[22] Balka E Doyle-Waters M Lecznarowicz D FitzGerald JM. Technology, governance and patient safety: systems issues in technology and patient safety. Int J Med Inform. (2007) 76:S35–47. 10.1016/j.ijmedinf.2006.05.046 16997620

[23] Amoore JN. A structured approach for investigating the causes of medical device adverse events. J Med Eng. (2014) 2014(1):314138. 10.1155/2014/314138 27006931 PMC4782710

[24] Arad D Finkelstein A Rozenblum R Magnezi R. Perceptions of surgical never events among interdisciplinary clinicians: implications of a qualitative study for practice: mental models and never events. Collegian. (2023) 30(2):321–6. 10.1016/j.colegn.2022.09.012

[25] Arad D Finkelstein A Rozenblum R Magnezi R. Patient safety and staff psychological safety: a mixed methods study on aspects of teamwork in the operating room. Front Public Health. (2022) 10:1060473. 10.3389/fpubh.2022.1060473 36620282 PMC9816421

[26] Braun V Clarke V. Using thematic analysis in psychology. Qual Res Psychol. (2003) 3(2):77–101. 10.1191/1478088706qp063oa

[27] Braun V Clarke V. One size fits all? What counts as quality practice in (reflexive) thematic analysis? Qual Res Psychol. (2021) 18(3):328–52. 10.1080/14780887.2020.1769238

[28] Hennink MM Kaiser BN Marconi VC. Code saturation versus meaning saturation: how many interviews are enough? Qual Health Res. (2017) 27(4):591–608. 10.1177/1049732316665344 27670770 PMC9359070

[29] Corbin J Strauss A. Grounded theory research : procedures, canons and evaluative criteria. Z Soziol. (1990) 19(6):418–27. 10.1515/zfsoz-1990-0602

[30] Gioia DA Corley KG Hamilton AL. Seeking qualitative rigor in inductive research: notes on the gioia methodology. Organ Res Methods. (2013) 16(1):15–31. 10.1177/1094428112452151

[31] Tong A Sainsbury P Craig J. Consolidated criteria for reporting qualitative research (COREQ): a 32-item checklist for interviews and focus groups. Int J Qual Health Care. (2007) 19(6):349–57. 10.1093/intqhc/mzm042 17872937

[32] Vincent C Amalberti R. Safer Healthcare: Strategies for the Real World. Cham: Springer Nature (2016). p. 1–170. 29465922

[33] Hewitt T Chreim S Forster A. Sociocultural factors influencing incident reporting among physicians and nurses: understanding frames underlying self-and peer-reporting practices. J Patient Saf. (2017) 13(3):129–37. 10.1097/PTS.0000000000000130 25119783

[34] World Health Organization. Medical Devices: Managing the Mismatch. Geneva: World Health Organization (2010).

[35] Lawton R Parker D. Barriers to incident reporting in a healthcare system. BMJ Qual Saf. (2002) 11(1):15–8. 10.1136/qhc.11.1.15 PMC1743585 12078362

[36] Sujan MA Spurgeon P Cooke MW. Translating tensions into safe practices through dynamic trade-offs : the secret second handover. Resilient Health Care. (2017) 2:41–52.

[37] Niv Y Kagan I Arad D Avrahami R Tal Y. Confidentiality of adverse events investigations – the present status and what is needed. Harefuah. (2022) 161(11):701–5. Hebrew. PMID: 36578242. 36578242

[38] Reason J. Human error: models and management. Br Med J. (2000) 320(7237):768–70. 10.1136/bmj.320.7237.768 10720363 PMC1117770

[39] Reason AJ. The contribution of latent human failures to the breakdown of Complex systems. Philos Trans R Soc Lond B Biol Sci. (1990) 327(1241):475–84. 10.1098/rstb.1990.0090 1970893

[40] Howell AM Burns EM Bouras G Donaldson LJ Athanasiou T Darzi A. Can patient safety incident reports be used to compare hospital safety? Results from a quantitative analysis of the English national reporting and learning system data. PLoS One. (2015) 10(12):e0144107. 10.1371/journal.pone.0144107 26650823 PMC4674095

[41] Dekker S. Drift Into Failure: From Hunting Broken Components to Understanding Complex Systems. London: CRC press (2016).

[42] Leape LL. Reporting of adverse events. N Engl J Med. (2002) 347(20):1633–8. 10.1056/NEJMNEJMhpr011493 12432059

[43] Espin S Lingard L Baker GR Regehr G. Persistence of unsafe practice in everyday work: an exploration of organizational and psychological factors constraining safety in the operating room. BMJ Qual Saf. (2006) 15(3):165–70. 10.1136/qshc.2005.017475 PMC2464856 16751464

[44] Edmondson A. Psychological safety and learning behavior in work teams. Adm Sci Q. (1999) 44(2):350–83. 10.2307/2666999

[45] Kaldjian LC Forman-Hoffman VL Jones EW Wu BJ Levi BH Rosenthal GE. Do faculty and resident physicians discuss their medical errors? J Med Ethics. (2008) 34(10):717–22. 10.1136/jme.2007.023713 18827101

[46] Jones A Kelly D. Whistle-blowing and workplace culture in older peoples' Care: qualitative insights from the healthcare and social care workforce. Sociol Health Illn. (2014) 36(7):986–1002. 10.1111/1467-9566.12137 24717014

[47] Weaver SJ Lubomksi LH Wilson RF Pfoh ER Martinez KA Dy SM. Promoting a culture of safety as a patient safety strategy: a systematic reviewe. Ann Intern Med. (2013) 158(5_Part_2):369–74. 10.7326/0003-4819-158-5-201303051-00002 23460092 PMC4710092

[48] Kagan I Barnoy S. Organizational safety culture and medical error reporting by Israeli nurses. J Nurs Scholarsh. (2013) 45(3):273–80. 10.1111/jnu.12026 23574516

[49] McGee RG Webster AC Rogerson TE Craig JC. Medical device regulation in Australia: safe and effective? Med J Aust. (2012) 196(4):256–60. 10.5694/mja11.11261 22409692

[50] Waring JJ. Beyond blame: cultural barriers to medical incident reporting. Soc Sci Med. (2005) 60(9):1927–35. 10.1016/j.socscimed.2004.08.055 15743644

[51] Badnjević A Pokvić LG Deumić A Bećirović LS. Post-market surveillance of medical devices: a review. Technol Health Care. (2022) 30(6):1315–29. 10.3233/THC-220284 35964220

[52] Damkjær M Elkjær M Hróbjartsson A Schroll JB. Scoping review on regulation, implementation and postmarket surveillance of medical devices. PLoS One. (2025) 20(5):e0325250. 10.1371/journal.pone.0325250 40445965 PMC12124561

[53] Amaral C Paiva M Rodrigues AR Veiga F Bell V. Global regulatory challenges for medical devices: impact on innovation and market access. Appl Sci. (2024) 14(20):9304. 10.3390/app14209304

[54] Choi SJ Choi S Park S Nam KC Jang HJ Kim JK The application study of harmonization code in medical device adverse event reporting. BMC Health Serv Res. (2024) 24(1):1402. 10.1186/s12913-024-11885-1 39543640 PMC11562707

[55] Hochreiter-Hufford A Gatz J Griggs AM Schoch RD Birmingham KM Frederick C Real-world data to support post-market safety and performance of embolization coils: evidence generation from a medical device manufacturer and data institute partnership. BMC Med Inform Decis Mak. (2024) 24(1):263. 10.1186/s12911-024-02659-0 39300415 PMC11414114

[56] Sun J Pan J Jin Y Zhang Q Lv Y Feng J. Establishment of a medical device adverse event management system for hospitals. BMC Health Serv Res. (2022) 22(1):1406. 10.1186/s12913-022-08830-5 36419040 PMC9685867

[57] Amarneh BH Al Nobani F. The influence of physician-nurse collaboration on patient safety culture. Heliyon. (2022) 8(9):e10649. 10.1016/j.heliyon.2022.e10649 36164521 PMC9508511

[58] Dietl JE Derksen C Keller FM Lippke S. Interdisciplinary and interprofessional communication intervention: how psychological safety fosters communication and increases patient safety. Front Psychol. (2023) 14:1164288. 10.3389/fpsyg.2023.1164288 37397302 PMC10310961

[59] Andrews G Davis KJ Govind N MacLellan A Kerrison-Watkin G Patel H. Learning together, responding together: interprofessional learning enhancing emergency services collaboration. Health Educ Pract. (2025) 8(1):1–19. 10.33966/hepj.8.1.20380

[60] Smit C Peddle M. Experiences and perceptions of registered nurses who work in acute care regarding incident reporting: a scoping review. Healthcare. (2025) 13(11):1250. MDPI. 10.3390/healthcare13111250 40508864 PMC12155484

[61] Lottes AE Cavanaugh KJ Chan YYF Devlin VJ Goergen CJ Jean R Navigating the regulatory pathway for medical devices—a conversation with the FDA, clinicians, researchers, and industry experts. J Cardiovasc Transl Res. (2022) 15(5):927–43. 10.1007/s12265-022-10232-1 35288821 PMC8920055

[62] Creswell JW Poth CN. Qualitative Inquiry and Research Design: Choosing Among Five Approaches. Thousand Oaks, CA: Sage Publications (2016).

