# Supplementary material for: Challenges and dynamics in reporting medical device incidents: a qualitative study
Source: Front Health Serv. 2025 Dec 10;5:1720494. doi: 10.3389/frhs.2025.1720494 (PMC12727643; doi:10.3389/frhs.2025.1720494)
Supplement: Supplementary file 1 [file Table1.docx]

**Supplementary Material 1. Interview guides**

**Interview Guide for Participants from Healthcare Institutions**
(e.g., clinical staff, risk managers, laboratory directors, hospital administrators, and biomedical engineering personnel)

Hello,

First, thank you very much for taking the time to meet with me.

My name is Meital Mishali, and I am a doctoral student in the School of Public Health at the University of Haifa. My research focuses on “Factors related to the reporting of medical device adverse events – perceptions among healthcare professionals and medical device companies.”

This study is being conducted through in-depth interviews with stakeholders involved in medical device adverse event reporting, including healthcare professionals and representatives of medical device companies.

The purpose of this interview is to explore how adverse events related to medical devices are reported in healthcare institutions, medical device companies, and the Ministry of Health; to examine the unique characteristics of such events; and to gain insights into the interactions between different stakeholders, as well as perceptions and barriers to reporting.

Your input will greatly contribute to a deeper understanding of the process and help promote more effective reporting. The intention is to hold an open and candid discussion on this topic, and your participation is highly valued.

The interview will be conducted online via Zoom. With your permission, I will record the conversation so that your ideas and perspectives can be documented as accurately as possible, without the need for me to take notes during the session. The recording will be stored on my personal, password-protected computer. Confidentiality of your responses is assured, and the recording will be deleted once it has been transcribed. Any personal information about you will remain strictly confidential and will not be shared with any third party.

There are no right or wrong answers—only your views matter. You may choose to withdraw from the interview at any stage.

Thank you again for agreeing to participate in this interview.

**Medical Device Adverse Events – Knowledge**

- What is your definition of an adverse event, and what is your definition of a medical device adverse event? Please provide an example.
- In your opinion, what is the difference between a medical device adverse event and a medical device malfunction?
- Can you describe the process of reporting medical device adverse events in your workplace? Please outline the reporting chain.

**Reporting Policies in Your Healthcare Institution**

- Please describe the formal and informal policies for reporting medical device adverse events in your workplace. Is there an established procedure for reporting such events?
- In your opinion, are all adverse events that occur in the department reported to risk management and subsequently forwarded to the Ministry of Health?
- Please describe how decisions are made in your workplace regarding whether or not to report an adverse event (to risk management or to the authorities). Who is involved in this process and in the decision-making?
- In your view, what interests are involved in the decision whether to report or not to report?

**Skills**

- In your opinion, is it more difficult to identify a medical device adverse event compared to another type of adverse event? Why?
- When you encounter a medical device adverse event in your work, can you describe the technical difficulties involved in reporting it?
- What do you think are the main causes of medical device adverse events (e.g., device malfunction, user error, clinical complication, lack of training or proper instruction on device use, incorrect installation)? Which of these do you think are easier to report? (For a healthcare professional / for a medical device company representative).

**Motivation and Goals**

- Please describe your motivation, or the motivation of other healthcare institution staff, to report a medical device adverse event.
- Are there any incentives for reporting a medical device adverse event?

**Barriers to Reporting**

- In your opinion, what are the reasons (challenges, barriers) that may lead to underreporting? (e.g., fear of blame, punishment, damage to career).
- How do time constraints affect your reporting of medical device adverse events?

**Social Influence (Norms)**

- Do you think medical device adverse events are reported by employees/colleagues in your workplace?
- How is reporting perceived by supervisors in your workplace—(as an opportunity for learning, as a reason for punishment)? Do they encourage reporting adverse events, or do they prefer to avoid it? What are the reasons for avoiding formal reporting?
- How would you describe your workplace’s approach to awareness and encouragement of reporting, such as through training or reminders? Or are no resources dedicated to this?
- Have you encountered resistance to reporting medical device adverse events? Please describe.

**Perceptions of the Usefulness of Reporting**

- What are the consequences of reporting a medical device adverse event (both positive and negative—including personal consequences)?
- What is your opinion about the usefulness or lack of usefulness of reporting an adverse event to the Ministry of Health and/or to the medical device company?

**Perceptions of Regulation**

- Are you familiar with the regulatory requirements for reporting medical device adverse events in Israel? What are they? Are they clear?
- In your opinion, what is the role of regulation in the field of adverse event reporting? What are your expectations?
- Do you think the regulatory framework in Israel regarding reporting is adequate? In your opinion, what could be improved in the reporting process or in the regulation itself?

**Interaction between Stakeholders: Medical Device Companies, Healthcare Institutions, and the Ministry of Health**

- In your opinion, is there collaboration between medical device companies and healthcare institutions?
- What interests do you think exist between the healthcare institution, as the user of medical devices, and the medical device company in relation to adverse events?
- How do the different stakeholders (the healthcare institution, the Ministry of Health, the medical device company [importer/manufacturer]) work together when handling an adverse event? Is there an established framework for collaboration?

**Personal Experience**

- Have you been involved in medical device adverse events and in their reporting? Please provide an example.

**Personal Background Information and Employment Status of Interviewee**

| **Category** | **Details** |
| --- | --- |
| **--- Personal Characteristics ---** |  |
| Gender |  |
| Year of Birth |  |
| Country of Birth |  |
| Ethnic Background | Jewish / Muslim / Christian / Druze |
| City of Residence |  |
| **--- Professional Characteristics ---** |  |
| Years of Professional Experience |  |
| Highest Level of Education | B.A. / M.A. / Ph.D. / M.D. / Other: _______ |
| **--- Employment Status ---** |  |
| Place of Employment |  |
| **Position** |  |
| Employed in a managerial role | Yes / No |
| Physician | Yes / No |
| Nurse | Yes / No |
| Other (Position) |  |
| Job Description |  |
| Specialization |  |
| Department |  |
| Years in Current Position |  |
| Work with Medical Devices on a | Daily / Weekly / Rare basis |
| Medical Devices Used in the Department |  |

**Interview Guide for Participants from Medical Device Companies**
*(e.g., chief executives, regulatory affairs managers, field representatives, and application specialists)*

**Interview Protocol for Employees in Medical Device Companies**

Hello,
First, thank you very much for taking the time to meet with me.

My name is Meital Mishali, and I am a doctoral student in the School of Public Health at the University of Haifa. My research focuses on *“Factors related to the reporting of medical device adverse events – perceptions among healthcare professionals and medical device companies.”*

This study is being conducted through in-depth interviews with stakeholders involved in medical device adverse event reporting, including healthcare professionals and representatives of medical device companies.

The purpose of this interview is to explore how adverse events related to medical devices are reported in healthcare institutions, in medical device companies, and at the Ministry of Health; to examine the unique characteristics of such events; and to gain insights into the interactions between different stakeholders, as well as perceptions and barriers to reporting.

Your input will greatly contribute to a deeper understanding of the process and help promote more effective reporting. The intention is to hold an open and candid discussion on this topic, and your participation is highly valued.

The interview will be conducted online via Zoom. With your permission, I will record the conversation so that your ideas and perspectives can be documented as accurately as possible, without the need for me to take notes during the session. The recording will be stored on my personal, password-protected computer. Confidentiality of your responses is assured, and the recording will be deleted once it has been transcribed. Any personal information about you will remain strictly confidential and will not be shared with any third party.

There are no right or wrong answers—only your views matter. You may choose to withdraw from the interview at any stage.

Thank you again for agreeing to participate in this interview.

**Medical Device Adverse Events – Knowledge**

- Please describe the process of reporting medical device adverse events in your workplace.
- What is your definition of an adverse event, and what is your definition of a medical device adverse event? Can you provide examples of such events?

**Reporting Policy in Your Company**

- Please describe how healthcare institutions report adverse events to your company (e.g., by phone, email, face-to-face), and outline the reporting chain—from the occurrence of an event in the healthcare institution, through the report to your company, and onward to your supervisors and the authorities. In your response, please specify whether there is a formal reporting procedure, a reporting form, or the use of dedicated reporting systems or platforms.
- Please describe the formal and informal policies for reporting medical device adverse events in your workplace (to the manufacturer / to the Ministry of Health). Is there an established reporting procedure?
- In your opinion, are all adverse events reported to your company (by customers) subsequently forwarded to the authorities (the Ministry of Health)? Which ones are, and which are not?
- Please describe how decisions are made in your company regarding whether or not to report an adverse event to the Ministry of Health. Who is involved in this process and in the decision-making?
- In your view, what interests are involved in the decision whether to report or not to report an event to the Ministry of Health?

**Skills**

- In your opinion, what is the difference between a medical device adverse event and a medical device malfunction?
- When you encounter a medical device adverse event in your work, can you describe the technical difficulties in reporting it (within your company—to your supervisor, to the authorities)?
- Do you think it is more difficult to identify a medical device adverse event compared to another type of adverse event? Why?
- What do you think are the main causes of medical device adverse events (e.g., device malfunction, user error, clinical complication, lack of training or proper instruction on device use, incorrect installation)? Which of these do you think are easier to report? (For a healthcare professional / for a medical device company representative).

**Motivation and Goals**

- Please describe your motivation to report a medical device adverse event that was forwarded to you from a healthcare institution (to your supervisor / to the authorities).
- Are there any incentives for reporting a medical device adverse event?
- Do you have other tasks or objectives that may interfere with reporting medical device adverse events?

**Barriers to Reporting**

- In your opinion, what are the reasons (challenges, barriers) that may lead to underreporting? (e.g., fear of blame, punishment, damage to career).
- How do time constraints affect your reporting of medical device adverse events?

**Social Influence (Norms)**

- Do you think medical device adverse events are reported by employees/colleagues in your company?
- How is reporting perceived by supervisors in your company—(as an opportunity for learning, as a reason for punishment)? Do they encourage reporting adverse events to the manufacturer/authorities, or do they prefer to avoid it? What are the reasons for avoiding formal reporting?
- How would you describe your company’s approach to awareness and encouragement of reporting, such as through training or reminders? Or are no resources dedicated to this?
- Have you encountered resistance to reporting medical device adverse events in your workplace? Please describe.

**Perceptions of the Usefulness of Reporting**

- What are the consequences of reporting a medical device adverse event to the authorities/manufacturer (both positive and negative—including personal consequences)?
- What is your opinion about the usefulness or lack of usefulness of reporting an adverse event to the authorities/manufacturer?

**Perceptions of Regulation**

- In your opinion, are the regulatory requirements for reporting medical device adverse events in Israel clear? What do you think could be improved?
- What do you see as the role of regulation in the field of adverse event reporting? What are your expectations?

**Interaction between Stakeholders: Medical Device Companies, Healthcare Institutions, and the Ministry of Health**

- How would you describe the level of collaboration between the Ministry of Health and medical device companies?
- What interests do you think exist between healthcare institutions, as users of medical devices, and medical device companies regarding adverse events related to marketed devices?
- How do the different stakeholders (healthcare institutions, the Ministry of Health, medical device companies [importer/manufacturer]) work together when handling an adverse event? Is there an established framework for collaboration?

**Personal Experience**

- Have you been involved in reporting a medical device adverse event to your supervisor / to the manufacturer / to the authorities? Please provide an example.

**Personal Background Information and Employment Status of Interviewee**

| **Category** | **Details** |
| --- | --- |
| **--- Personal Characteristics ---** |  |
| Gender |  |
| Year of Birth |  |
| Country of Birth |  |
| Ethnic Background | Jewish / Muslim / Christian / Druze |
| City of Residence |  |
| **--- Professional Characteristics ---** |  |
| Years of Professional Experience |  |
| Highest Level of Education | B.A. / M.A. / Ph.D. / M.D. / Other:______ |
| **--- Employment Status ---** |  |
| Place of Employment |  |
| **Position** |  |
| Managerial Role | Yes / No |
| Regulatory Affairs | Yes / No |
| Sales | Yes / No |
| Application Specialist | Yes / No |
| Service | Yes / No |
| Other (Position) |  |
| Job Description |  |
| Department in the Company |  |
| Years in Current Position |  |
| Employment Status | Part-time / Full-time |
| Medical Devices Worked With |  |
