# Supplementary material for: Challenges and dynamics in reporting medical device incidents: a qualitative study
Source: Front Health Serv. 2025 Dec 10;5:1720494. doi: 10.3389/frhs.2025.1720494 (PMC12727643; doi:10.3389/frhs.2025.1720494)
Supplement: Supplementary file 2 [file Table2.docx]

**Supplementary Material 2. Consolidated criteria for reporting qualitative studies (COREQ): 32-item checklist**

| **No.** | **Item** | **Guide questions / description** | | **Page** |
| --- | --- | --- | --- | --- |
| **Domain 1: Research team and reflexivity**  ***Personal characteristics*** | | | | |
| 1. | Interviewer / facilitator | Which author/s conducted the interviews or focus group? | Meital Mishali | Page 16 – Author Contributions |
| 2. | Credentials | What were the researcher’s credentials? E.g. PhD, MD | Meital Mishali = B.Sc, MSC, PhD student  Nadav Sheffer = PhD  Maya Negev = PhD | Not in manuscript |
| 3. | Occupation | What was their occupation at the time of the study? | Meital Mishali = PhD student  Nadav Sheffer and Maya Negev = Academic faculty members | Not in manuscript |
| 4. | Gender | Was the researcher male or female? | Meital Mishali and Maya Negev = Females  Nadav Sheffer = Male | Not in manuscript |
| 5. | Experience and training | What experience or training did the researcher have? | Meital Mishali received training in qualitative research methods during her PhD studies. Maya Negev is an experienced qualitative researcher with multiple peer-reviewed publications using qualitative methodologies in public health and health systems research. Nadav Sheffer has no formal qualitative training; he is the former Director of the Medical Devices Division, with professional and academic expertise in medical devices and ongoing research in the field. | Not in manuscript |
| ***Relationship with participants*** | | | | |
| 6. | Relationship established | Was a relationship established prior to study commencement? | In most cases, no prior relationship existed between the interviewer and participants. A professional relationship existed only with some interviewees from the Ministry of Health. | Not in manuscript |
| 7. | Participant knowledge of the interviewer | What did the participants know about the researcher? E.g. personal goals, reasons for doing the research | They knew the interviewer was part of the study team conducting research on medical device adverse event reporting. | Supplementary Material 1 – Interview guide. |
| 8. | Interviewer characteristics | What characteristics were reported about the interviewer/facilitator? E.g. bias, assumptions, reasons and interests in the research topic | Meital Mishali works at the Ministry of Health in the Medical Device Division; however, she conducted the interviews in her role as a doctoral researcher. Interviewees were informed in advance, via email, about her role and the study objectives. Given her professional background, she was aware of potential assumptions and interests in the topic, and therefore took care to maintain a neutral and open approach during the interviews. | Not in manuscript |
| **Domain 2: study design**  ***Theoretical framework*** | | | | |
| 9. | Methodological orientation and theory | What methodological orientation was stated to underpin the study? E.g. grounded theory, discourse analysis, phenomenology, content analysis | The study was underpinned by a reflexive thematic analysis approach, based on data generated through semi-structured interviews. The analysis combined deductive codes derived from the interview guide with inductive codes that emerged from the data. | Page 4 – Methods, Data analysis |
| ***Participant selection*** | | | | |
| 10. | Sampling | How were participants selected? E.g. purposive, convenience, consecutive, snowball | Purposive sampling was applied to recruit key policymakers and managers from the Ministry of Health, hospitals, and medical device companies across Israel. Hospital participants were drawn from three types of institutions—government, private, and Clalit Health Services hospitals—to ensure broad representation. For industry, companies marketing diverse types of products were included. Snowball sampling was additionally employed to expand the sample through professional networks. | Page 4 – Methods, Sample selection and recruitment. |
| 11. | Method of approach | How were participants approached? E.g. face-to-face, telephone, mail, email | Face-to-face, email and telephone. | Not in manuscript |
| 12. | Sample size | How many participants were in the study? | **31 interviews were conducted.** | Page 3 -Methods, Approach |
| 13. | Non-participation | How many people refused to participate or dropped out? Reasons? | Two individuals who were approached declined or did not respond. There were no participants that dropped out of the study. | Not in manuscript |
| ***Setting*** | | | | |
| 14. | Setting of data collection | Where was the data collected? E.g. home, clinic, workplace | Face-to-face (2 workplaces, 1 home), Zoom (25), telephone (3). | Page 3 -Methods, Approach |
| 15. | Presence of non-participants | Was anyone else present besides the participants and researchers? | Only participant and researcher, except two company interviews where an additional employee was present*.* | Not in manuscript |
| 16. | Description of the sample | What are the important characteristics of the sample? E.g. demographic data, date | 31 participants: 4 Ministry of Health policymakers/managers; 17 hospital staff (physicians, nurses, risk managers, biomedical engineers, administrators) from government, private, and HMO hospitals; 10 company representatives (executives, regulatory affairs, application specialists). Gender: 15 women, 16 men (Table 1). | Table 1 |
| ***Data collection*** | | | | |
| 17 | Interview guide | Were questions, prompts, guides provided by the authors? Was it pilot tested? | Semi-structured interview guide, slightly adapted per stakeholder group, pilot tested (3 interviews) (see Appendix A). | Supplementary Material 1 – Interview guide. |
| 18. | Repeat interviews | Were repeat interviews carried out? If yes, how many? | No. | NA |
| 19. | Audio / visual recording | Did the research use audio or visual recording to collect the data? | Yes. Zoom interviews were audio–visually recorded; face-to-face and telephone interviews were audio-recorded only. | Page 3 -Methods, Approach |
| 20. | Field notes | Were field notes made during and/or after the interviews? | Yes | Not in manuscript |
| 21. | Duration | What was the duration of the interviews of focus groups? | Interviews lasted 30–60 minutes. | Page 3 -Methods, Approach |
| 22. | Data saturation | Was data saturation discussed? | Yes | Page 4 – Methods, Sample selection and recruitment. |
| 23. | Transcripts returned | Were transcripts returned to participants for comments and/or corrections? | No | NA |
| **Domain 3: analysis and findings**  ***Data analysis*** | | | |  |
| 24. | Number of data coders | How many data coders coded the data? | One (Meital Mishali) | Page 16 – Author Contributions |
| 25. | Description of the coding tree | Did authors provide a description of the coding tree? | No | NA |
| 26. | Derivation of themes | Were themes identified in advance or derived from the data? | Themes were identified through a combination of deductive coding based on the interview guide and inductive coding derived from the data. | Page 4 – Methods, Data analysis |
| 27. | Software | What software, if applicable, was used to manage the data? | ATLAS.ti software was used to manage, code, and analyze the interview transcripts. | Page 4 – Methods, Data analysis |
| 28. | Participant checking | Did participants provide feedback on the findings? | No | NA |
| ***Reporting*** | |  | |  |
| 29. | Quotations presented | Were participant quotations presented to illustrate the themes / findings?  Was each quotation identified? E.g. participant number | Yes  Yes | Pages 5–13 – Results |
| 30. | Data and findings consistent | Was there consistency between the data presented and the findings? | Yes | Pages 5–13 – Results |
| 31. | Clarity of major themes | Were major themes clearly presented in the findings? | 4 key themes were identified in the study and presented in the manuscript. All of the themes were discussed in the results section. | Pages 5–13 – Results |
| 32. | Clarity of minor themes | Is there a description of diverse cases or discussion of minor themes? | Yes. Diverse cases were presented to reflect differences across stakeholder groups. | Pages 5–13 – Results |

Reference:

Tong, A., Sainsbury, P., Craig, J. (2007) Consolidated criteria for reporting qualitative research (COREQ): a 32-item checklist for interviews and focus groups. International Journal Quality Health Care 19(6):349–357
